# Supplementary material for: Glycan-reactive antibodies isolated from human HIV-1 vaccine trial participants show broad pathogen cross-reactivity
Source: bioRxiv. 2025 Jan 20:2025.01.17.633475. Preprint. [Version 1] doi: 10.1101/2025.01.17.633475 (PMC11785028; doi:10.1101/2025.01.17.633475)
Supplement: 1 [file NIHPP2025.01.17.633475V1-supplement-1.pdf]

Figure S1

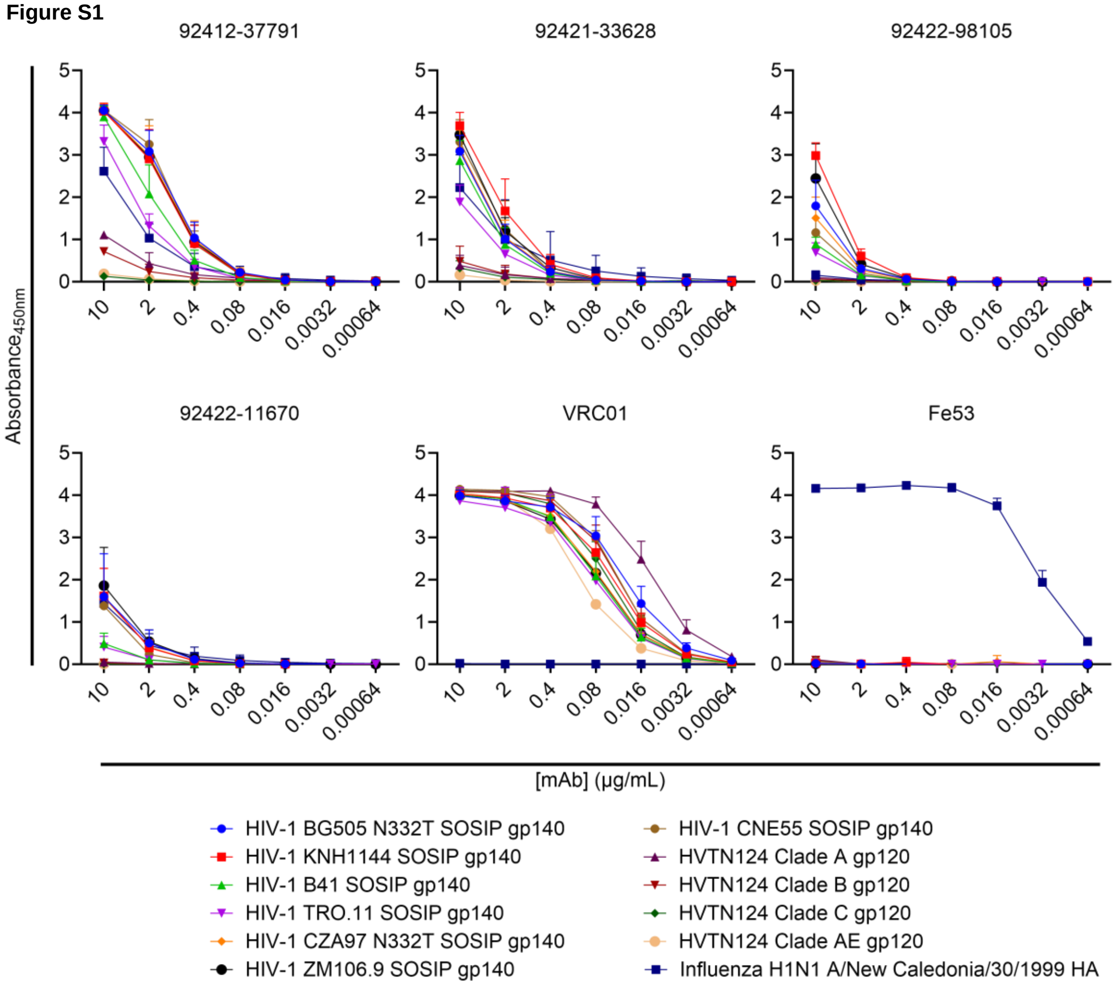

Figure S1. Characterization of HVTN124 mAbs by ELISA.
ELISA validation of HVTN124 mAbs displayed as full curves. ELISA 5-fold curves from a set of three repeats in duplicate are displayed for each of the four HVTN124 mAbs, along with the VRC01 and Fe53 mAb controls, against all 12 antigens used in the LIBRA-seq screening library.

Figure S2

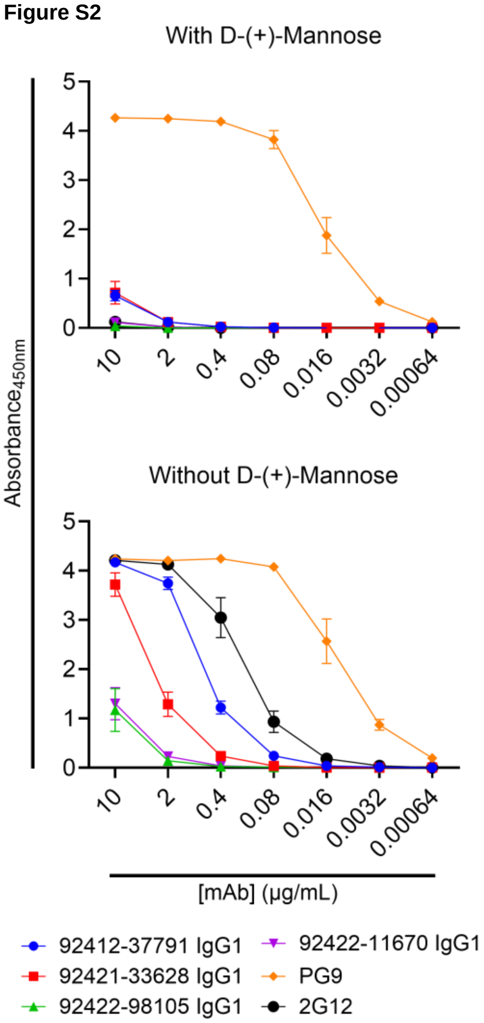

Figure S2. HVTN124 mAbs achieve broad reactivity via N-linked glycan recognition.
Antibody competition with and without 1M D-(+)-Mannose displayed as full curves. Absorbance at 450nm is listed on the Y-axis, while antibody concentration in μg/mL is listed on the X-axis. The four HVTN124 mAbs, along with the V3-glycan-reactive 2G12 and V1/V2-reactive PG9 control mAbs, were incubated with and without 1M D-(+)-Mannose against HIV-1 CNE55 SOSIP gp140. ELISA 5-fold curves for both conditions from a set of three repeats in duplicate are displayed.

Figure S3

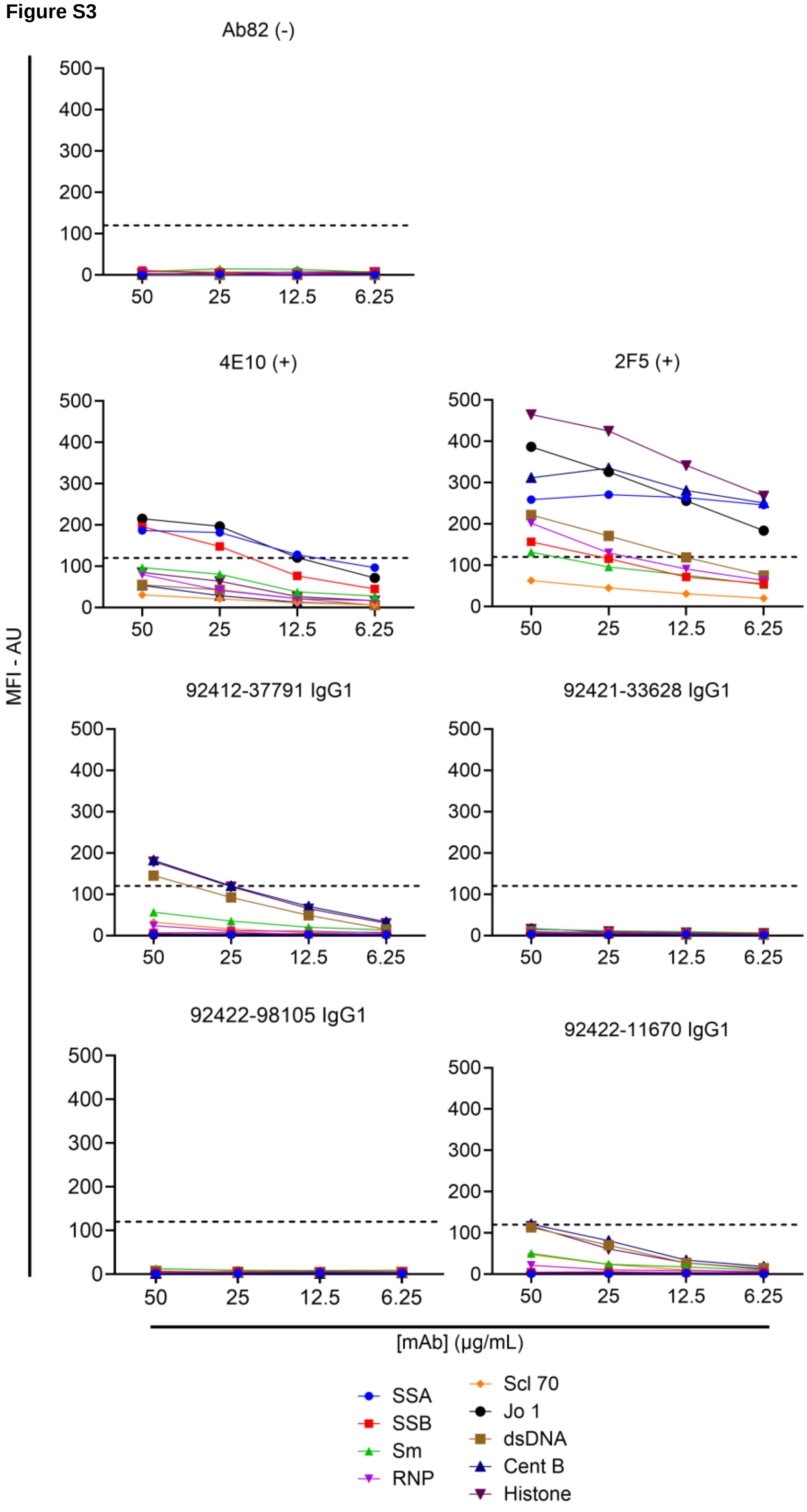

Figure S3. AtheNA autoreactivity analysis of HVTN124 mAbs.
Autoantigen reactivity against the AtheNA panel as full curves. HVTN124 mAbs were tested for autoreactivity against the AtheNA panel. MFI – AU is listed on the Y-axis, while antibody concentration in µg/mL is listed on the X-axis. Positive control antibodies included 4E10 and 2F5, while Ab82 was used as a negative control antibody. Values exceeding 120 MFI at 25 µg/mL for the AtheNA assay are considered positive. Negative MFI – AU values were transformed to zero.

Figure S4

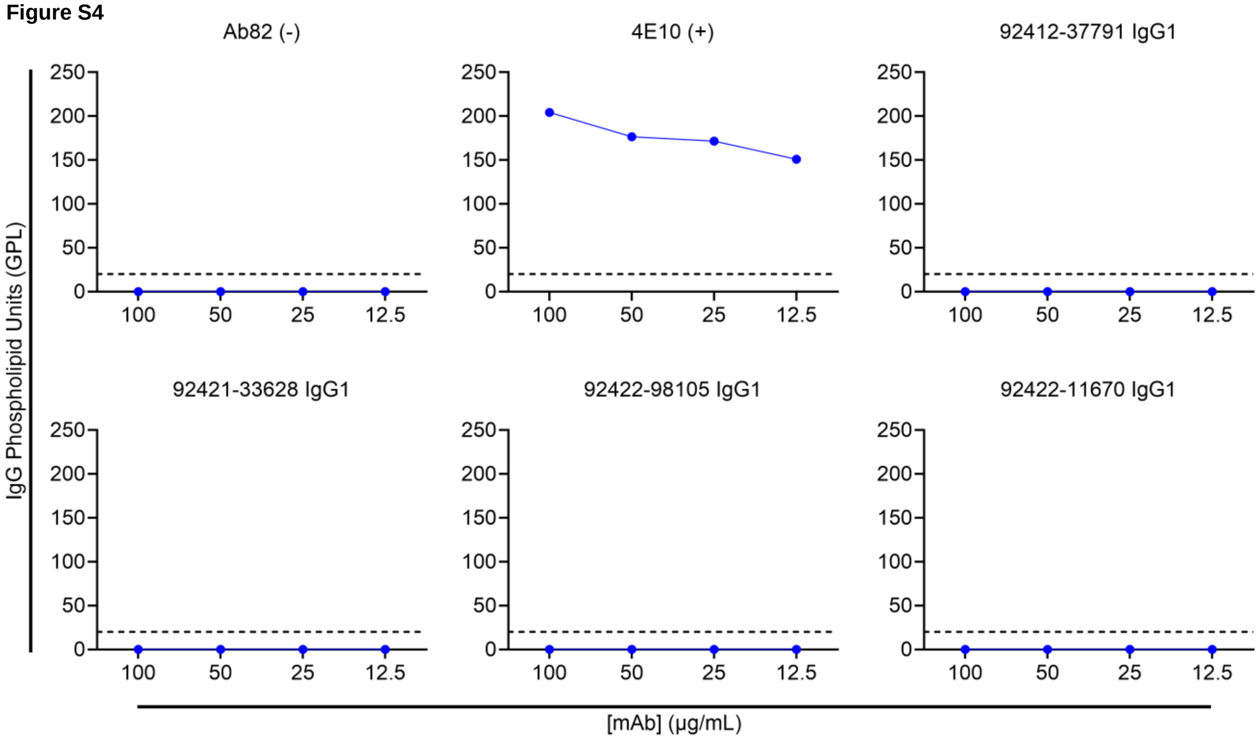

Figure S4. Cardiolipin autoreactivity analysis of HVTN124 mAbs.
Autoreactivity toward cardiolipin as full curves. IgG phospholipid units (GPL) is listed on the Y-axis, while antibody concentration in µg/mL is listed on the X-axis. 4E10 was used as a positive control antibody, while Ab82 was used as a negative control antibody. Values at or greater than 20 GPL at 50 µg/mL are considered positive. Negative GPL values were transformed to zero.

Figure S5

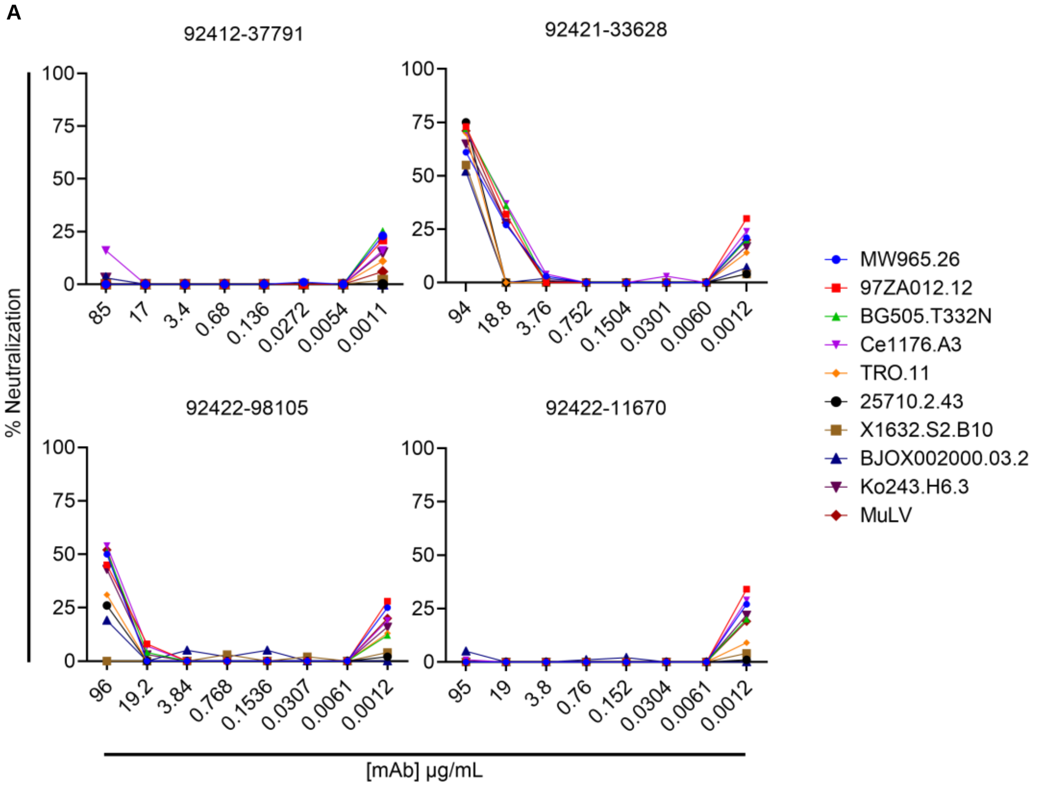

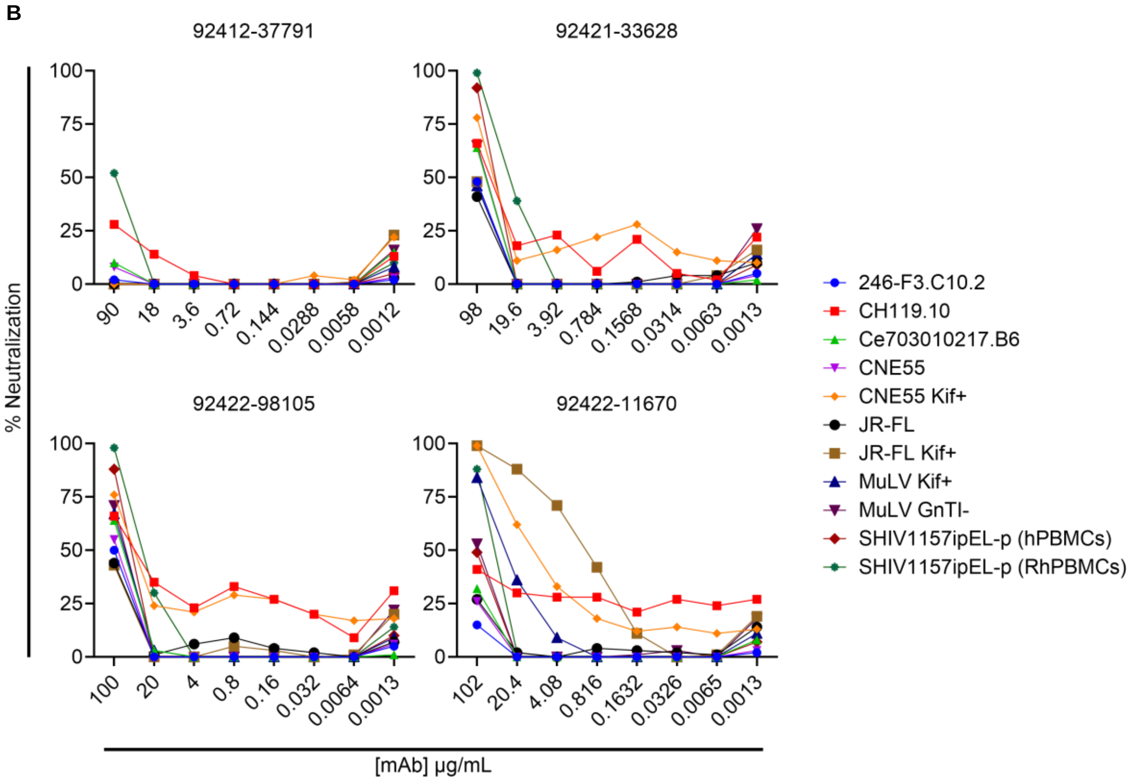

Figure S5. HIV-1 neutralization.
(A) HVTN124 mAbs vs. HIV-1 pseudovirus set 1. Lot 1 of HVTN124 mAbs vs. HIV-1 pseudovirus. Percent neutralization is listed on the Y-axis while antibody concentration in µg/mL is listed on the X-axis. Percent neutralization of the 5-fold curves are displayed for each of the four HVTN124 mAbs being tested against: MMW965.26, 97ZA012.12, BG505.T332N, Ce1176.A3, TRO.11, 25710.2.43, X1632.S2.B10, BJOX002000.03.2, Ko243.H6.3, and MuLV. Negative percent neutralization values were transformed to zero.
(B) HVTN124 mAbs vs. HIV-1 pseudovirus set 2. Percent neutralization is listed on the Y-axis while antibody concentration in µg/mL is listed on the X-axis. Percent neutralization of the 5-fold curves are displayed for each of the four HVTN124 mAbs being tested against: 246-F3.C10.2, CH119.10, Ce703010217.B6, CNE55, CNE55 Kif+, JR-FL, JR-FL Kif+, MuLV Kif+, MuLV GnTI-, SHIV1157ipEL-p (hPBMCs), and SHIV1157ipEL-p (RhPBMCs). Negative percent neutralization values were transformed to zero.

Figure S6

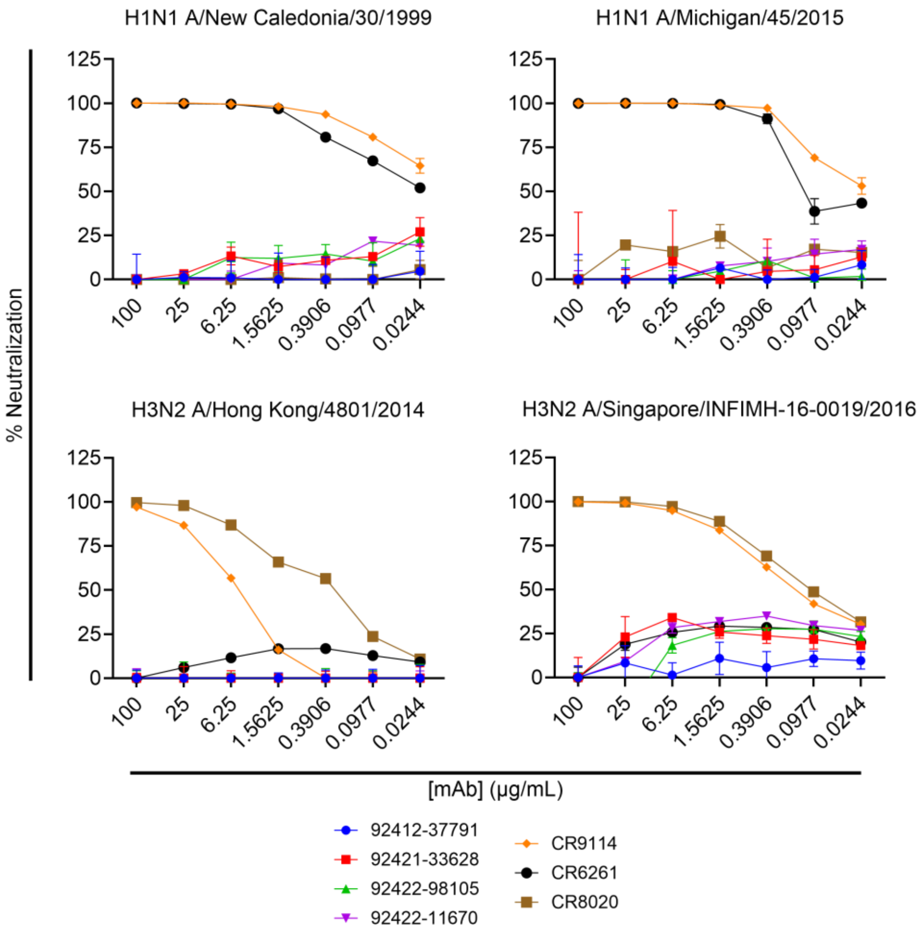

Figure S6. Influenza neutralization.

Neutralization of HVTN124 mAbs vs. H1N1 and H3N2 Influenza viruses as 4-fold curves. HVTN124 mAbs were tested for neutralizing activity against an influenza viral panel that included: H1N1 A/New Caledonia/30/1999, H1N1 A/Michigan/45/2015, H3N2 A/Singapore/INFIMH-16-0019/2016, and H3N2 A/Hong Kong/4801/2014. Of the control mAbs used, CR9114 is known to broadly neutralize influenza A and B viruses, while CR6261 targets group 1 influenza A viruses such as H1N1 isolates, and CR8020 targets group 2 influenza A viruses such as H3N2 isolates. The percent neutralization is listed on the Y-axis, while the antibody concentrations in µg/mL is listed on the X-axis. Negative percent neutralization values were transformed to zero.

Figure S7

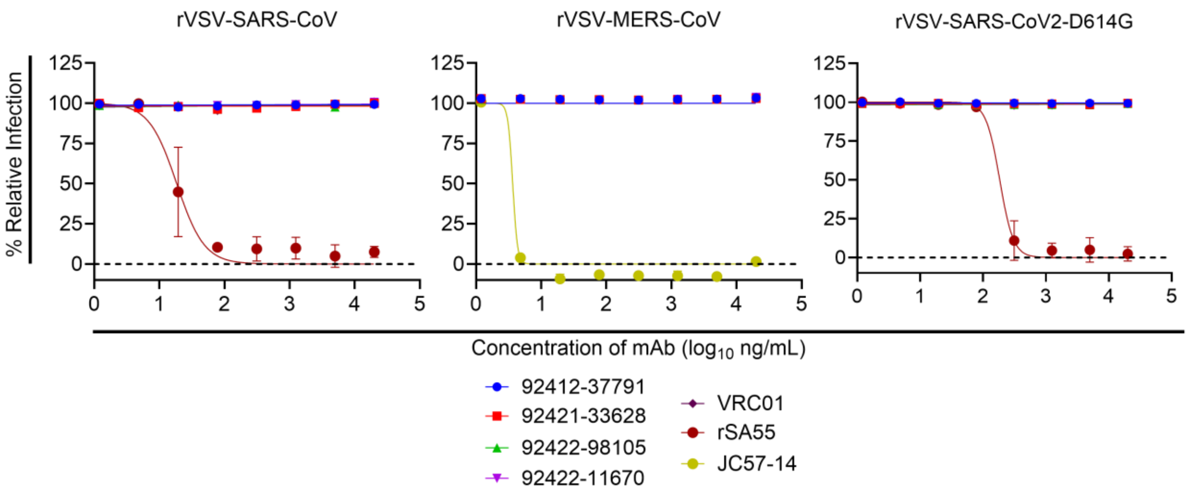

Figure S7. Coronavirus neutralization.
Coronavirus neutralization as 4-fold curves. HVTN124 mAbs were screened for neutralizing activity against rVSV-SARS-CoV-2 D614G, rVSV-MERS-CoV, and rVSV-SARS-CoV. Positive controls included rSA55 for rVSV-SARS-CoV-2 D614G and rVSV-SARS-CoV, while JC57-14 was used for rVSV-MERS-CoV. VRC01 was also included as a negative control mAb. The percent infectivity is listed on the Y-axis, while the antibody concentrations in µg/mL mAb is listed on the X-axis. Data are mean ± standard deviations (SD) of technical triplicates from a representative experiment repeated twice.
